# Medical Assistance in Dying in patients with advanced cancer and their caregivers: a mixed methods longitudinal study protocol

**DOI:** 10.1186/s12904-021-00793-4

**Published:** 2021-07-21

**Authors:** Madeline Li, Gilla K. Shapiro, Roberta Klein, Anne Barbeau, Anne Rydall, Jennifer A. H. Bell, Rinat Nissim, Sarah Hales, Camilla Zimmermann, Rebecca K. S. Wong, Gary Rodin

**Affiliations:** 1 Department of Supportive Care, Princess Margaret Cancer Centre, University Health Network, 620 University Avenue, 12th Floor, Toronto, Ontario M5G 2C1 Canada; 2 Department of Psychiatry, Faculty of Medicine, University of Toronto, Toronto, Ontario Canada; 3 Global Institute of Psychosocial, Palliative and End-of-Life Care (GIPPEC), University of Toronto and Princess Margaret Cancer Centre, Toronto, Ontario Canada; 4 Joint Centre for Bioethics, University of Toronto, Toronto, Ontario Canada; 5 Department of Medicine, University of Toronto, Toronto, Ontario Canada; 6 Department of Radiation Oncology, University of Toronto, Toronto, Ontario Canada

**Keywords:** Cancer, Depression, Desire for hastened death, Euthanasia, Medical assistance in dying, Medical communication, Palliative care, Distress, Assisted dying, Will to live

## Abstract

**Background:**

The legal criteria for medical assistance in dying (MAiD) for adults with a grievous and irremediable medical condition were established in Canada in 2016. There has been concern that potentially reversible states of depression or demoralization may contribute to the desire for death (DD) and requests for MAiD. However, little is known about the emergence of the DD in patients, its impact on caregivers, and to what extent supportive care interventions affect the DD and requests for MAiD. The present observational study is designed to determine the prevalence, predictors, and experience of the DD, requests for MAiD and MAiD completion in patients with advanced or metastatic cancer and the impact of these outcomes on their primary caregivers.

**Methods:**

A cohort of patients with advanced or metastatic solid tumour cancers and their primary caregivers will be recruited from a large tertiary cancer centre in Toronto, Ontario, Canada, to a longitudinal, mixed methods study. Participants will be assessed at baseline for diagnostic information, sociodemographic characteristics, medical history, quality of life, physical and psychological distress, attitudes about the DD and MAiD, communication with physicians, advance care planning, and use of psychosocial and palliative care interventions. Measures will subsequently be completed every six months and at the time of MAiD requests. Quantitative assessments will be supplemented by qualitative interviews in a subset of participants, selected using quota sampling methods.

**Discussion:**

This study has the potential to add importantly to our understanding of the prevalence and determinants of the DD, MAiD requests and completions in patients with advanced or metastatic cancer and of the experience of both patients and caregivers in this circumstance. The findings from this study may also assist healthcare providers in their conversations about MAiD and the DD with patients and caregivers, inform healthcare providers to ensure appropriate access to MAiD, and guide modifications being considered to broaden MAiD legislation and policy.

## Background

Following the decision of the Supreme Court of Canada on February 6, 2015 to decriminalize medical assistance in dying (MAiD), [1] the Federal Parliament of Canada passed Bill C-14 on June 17, 2016 to establish the legal eligibility criteria for MAiD. These criteria included the presence of a serious and incurable medical condition, an advanced state of irreversible decline in capability, enduring and intolerable physical or psychological suffering, and a reasonably foreseeable natural death [2]. Bill C-14 excluded mature minors or those with mental illness as the sole underlying medical condition. Legislative review of these criteria are now underway, and the passage of Bill C-7 on March 17, 2021 removed the requirements of a "reasonably foreseeable natural death" [3, 4].

Over 21,500 people in Canada chose to end their lives through assisted dying in the first five years of MAiD, more than 67% of whom had advanced cancer [4]. The frequency of MAiD in Canada is increasing annually, [5] although the overall proportion of medically assisted deaths is in the middle of the international rates reported. These range from 0.3% of deaths in Oregon, United States, to 4.6% in the Netherlands [4, 6]. In 2019, MAiD deaths in Canada represented approximately 2.5% of all deaths, [4] and 6.3% of cancer-related deaths [4, 7].^1^

In 2007, we completed a longitudinal study of the desire for death (DD) in individuals with advanced cancer [8]. This early research confirmed that the DD occurs on a continuum from the passive wish that the end would come earlier than it would otherwise naturally occur, referred to as the desire for hastened death (DHD) or the wish to hasten death (WTHD), [9–11] to a more active desire to end life, reflected in requests for MAiD [12]. The DD is uncommon in patients with advanced cancer receiving active treatment, [8] but is reported in up to half of all individuals in palliative care, [13–15] and may paradoxically coexist with the will to live [16]. This paradox underlines the complexity of psychological states related to the DD and the importance of reflective conversations before action on such wishes is taken [17].

Research examining attitudes and correlates of the WTHD in patients with advanced cancer has largely been cross-sectional, retrospective or conducted in settings where assisted dying is not permitted [18–22]. In research conducted in palliative care settings, a transient WTHD was found in 11–55% of patients, while 3–20% reported a more pervasive and persistent wish to die [13]. In a Canadian survey of 377 cancer patients with a prognosis of less than six months, Wilson and colleagues [13] found that almost 70% reported no WTHD, more than 18% acknowledged a transient WTHD, and over 12% reported a clear and persistent WTHD on the Desire for Death Rating Scale [14]. These findings are consistent with our previous study in which more than 65% of patients with advanced cancer indicated they wanted to continue living regardless of the pain or suffering that their disease might cause, and only 1.2% reported a clear WTHD on the Schedule of Attitudes towards Hastened Death (≥10) [8]. This difference between the two studies is likely due to the Wilson study sampling patients closer to the end of life, whereas we studied ambulatory patients recruited from oncology clinics.

Studies have also found that the DD fluctuates widely over time, [8, 14, 23, 24] even during the last two weeks of life [25]. In a sample of almost one thousand American patients with advanced disease, Emanuel and colleagues found that more than 10% reported seriously considering assisted death for themselves. However, on follow-up two to six months later, approximately half had changed their minds, and an almost equal number who had not initially been interested in assisted death were then considering it [26].

**Fig. 1:**
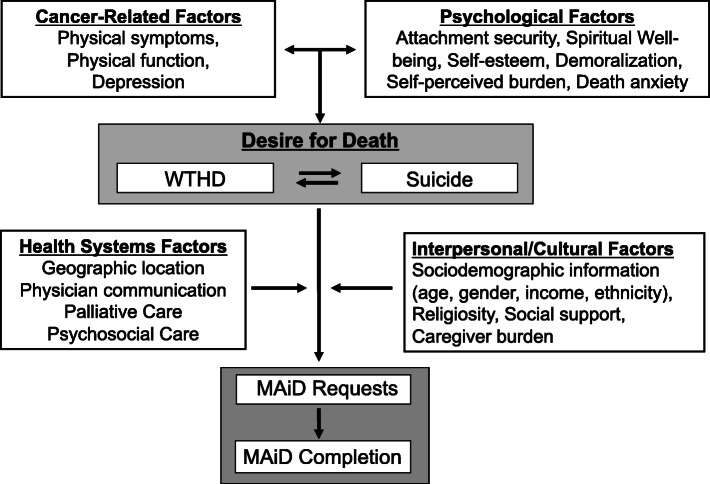
MAiD, Medical Assistance in Dying; WTHD, Wish to Hasten Death

The DD in patients with advanced cancer may arise from a complex interaction of factors, such as the experience or fear of uncontrolled physical or psychological suffering, the wish to maintain a sense of personal autonomy, or the desire to avoid burdening others (Fig. 1) [27, 28]. It has also been associated with pain, high disease burden, low self-esteem, poor spiritual well-being, attachment insecurity, being unmarried, younger age, and having a history of psychiatric illness [18, 25, 26, 29–32]. Some of these factors, such as those related to self-esteem, spiritual well-being, and attachment security, may be modifiable through specific psychotherapies, such as Managing Cancer and Living Meaningfully, [33] a brief, supportive-expressive intervention developed by our team and now being delivered internationally. Attachment security refers to an individual’s expectations and preferences regarding support from significant others in times of need and the capacity to make use of such support to relieve distress [34]. A secure attachment style, reflecting comfort with closeness and reliance on others, has been shown to protect against depression in cancer patients [35]. An avoidant attachment style, characterized by inflexible self-reliance, the need for personal control, and reluctance to accept support from others, has been associated in cancer patients with problematic relationships with oncologists [36] and requests for assisted dying [30, 37].

Physical symptom burden has been found to predict the DD [8, 26, 29] in patients with advanced cancer, although the motivation for MAiD in those who request and receive it is more often related to the loss of meaning, autonomy, and identity [19, 38, 39]. Some studies have found that religious observance and religious denomination have been linked to attitudes about assisted dying [40, 41]. The influence of social and demographic factors on MAiD requests also deserves further exploration. Research thus far indicates that MAiD completion is more common in those who are older, Caucasian, more highly educated, and more affluent [38, 39].

Dramatic changes have occurred over the past decade in Canada and throughout the world in public attitudes and expectations about death and dying, in access to psychosocial and palliative care for cancer patients, and, most recently, in the availability of MAiD. These changes may increase the likelihood that individuals with advanced disease and their caregivers will reflect upon and communicate the DD, engage in advance care planning, or consider actions to end their lives. While patients who request MAiD most often have access to specialized palliative care in settings where this is available, [4, 42, 43] the extent to which early palliative and supportive care interventions and empathic communication with healthcare providers about the goals of care at the end of life can affect the DD and requests for MAiD is unknown. Further, although caregivers are likely to be affected by patients’ DD and MAiD requests, [44–49] there is only limited research evaluating their experiences and support needs in these circumstances [20, 26, 48].

Caregivers typically experience a dramatic change in their daily lives and roles following the diagnosis of cancer or advanced disease in a loved one, related to the loss of family income [48] and the requirements for practical, social, and emotional support [47, 49]. They must often shoulder the physical and psychological burden of caregiving, while also facing the suffering and threat of mortality in their loved one, their own fear of facing the future alone, and the financial strain caused by the illness. Although decisions about assisted dying may have an impact on primary caregivers, we know relatively little about the impact of their distress on the emergence and persistence of the DD and MAiD requests in patients with advanced cancer [50]. Understanding the experience of caregivers in relation to MAiD is therefore an important area in need of further research.

Optimal end-of-life care for patients with advanced cancer requires healthcare providers to engage in conversations with those who express a DD and/or who request MAiD. Cross-sectional research has identified some factors associated with the DD and MAiD requests, but it is not possible from such studies to distinguish confounding from correlational and causal factors, or to determine the course of an individual’s desire for MAiD in the face of advanced cancer.

We will be conducting a longitudinal study to examine the prevalence, trajectory, predictors, and nature of the desire for MAiD in a geographic setting where MAiD is legally available. The goals of this mixed methods study are to estimate the prevalence of the DD in patients with advanced cancer; to understand the trajectory and contributors to the DD, MAiD requests and MAID completion; and to elucidate the experience and support needs of their primary caregivers. The findings of this study could inform the training of healthcare providers regarding conversations with patients with advanced cancer and their caregivers about goals of care at the end of life, the DD, and MAiD as well as psychotherapeutic conversations about the DD. This information will also support the review of legislation to ensure appropriate access to MAiD.

### Study aims

1. To determine in patients with advanced cancer:
  - The prevalence of the DD, MAiD requests, and MAiD completions.
  - The trajectory and predictors of the DD, MAiD requests, and MAiD completions.
2. To examine the predictors of distress in primary caregivers of patients with advanced cancer.

## Methods

### Study design

This research is a prospective, longitudinal, mixed methods study conducted with patients with advanced or metastatic cancer and their primary caregivers. This study was approved on September 2, 2020, by the University Health Network Research Ethics Board (UHN REB #18–6182), and all participants will provide informed consent. Any protocol amendments will be submitted to the UHN REB for approval, and important changes that could impact participants’ willingness to continue in the study will be communicated to them in a timely manner. Data collection is expected to begin by January 2021.

### Participants and setting

We plan to retain a longitudinal cohort of 600 patients with advanced or metastatic cancer and 300 of their primary caregivers recruited from the outpatient medical oncology clinics at the Princess Margaret Cancer Centre (PM), University Health Network, in Toronto, Canada’s largest comprehensive cancer treatment, education and research centre. This sample size is required to test our proposed regression model for predictors of MAiD requests and completions (Fig. 1), in which we will need at least 110 patients with at least two data points (baseline and one follow-up) [8], based on five to ten observations required for each of the 11 predictors included in our SEM [51]. Given that MAiD accounts for almost 5% of cancer deaths, and this represents 26% of MAiD requests at PM [39], we estimate ~ 115 subjects requesting MAiD and ~ 30 subjects receiving MAiD among 600 recruited patients.

To accommodate an anticipated 25% drop-out rate, we will recruit 800 patients and 400 primary caregivers over approximately 3.5 years for a final sample size of 600 patients and 300 primary caregivers. This is feasible given that PM provides care for approximately, 3200 Stage III or IV cancer patients per year, 60% of whom are expected to meet inclusion criteria; a conservative estimate is that 50% will provide informed consent, yielding approximately 960 eligible and consenting participants per year.

#### Participant recruitment and eligibility

Patients will be included if they: 1) are age 18 years or older; 2) are able to speak and read English sufficiently well to provide informed consent and complete questionnaires and/or interviews; and 3) have been diagnosed with advanced or metastatic solid tumour cancers of any type. Exclusion criteria will include: 1) significant cognitive impairment documented in their medical record or indicated by a score of < 20 on the Short Orientation-Memory-Concentration Test (SOMC); and 2) lack of sufficient proficiency in the English language to provide informed consent and complete the questionnaires and/or interviews. Trained research personnel will conduct the informed consent discussion with eligible and interested patients. Eligible patients who decline to participate will be asked their permission to allow the research team to document basic demographics to allow for later assessment of generalizability. Participants who begin the study will be considered withdrawn if they are later unable to participate due to a deterioration in cognitive functioning or physical capacity.

Individuals identified by participants as their primary caregivers will be approached by research personnel for recruitment if they are: 1) age 18 years or older; and 2) able to speak and read English sufficiently well to provide informed consent and complete questionnaires and/or interviews. A primary caregiver may be the spouse or partner of the patient, a relative or other family member, or a close friend. Caregivers may continue to participate in this study even if the patient participant subsequently withdraws from the study.

### Study measures

#### Questionnaire package

**Fig. 2:**
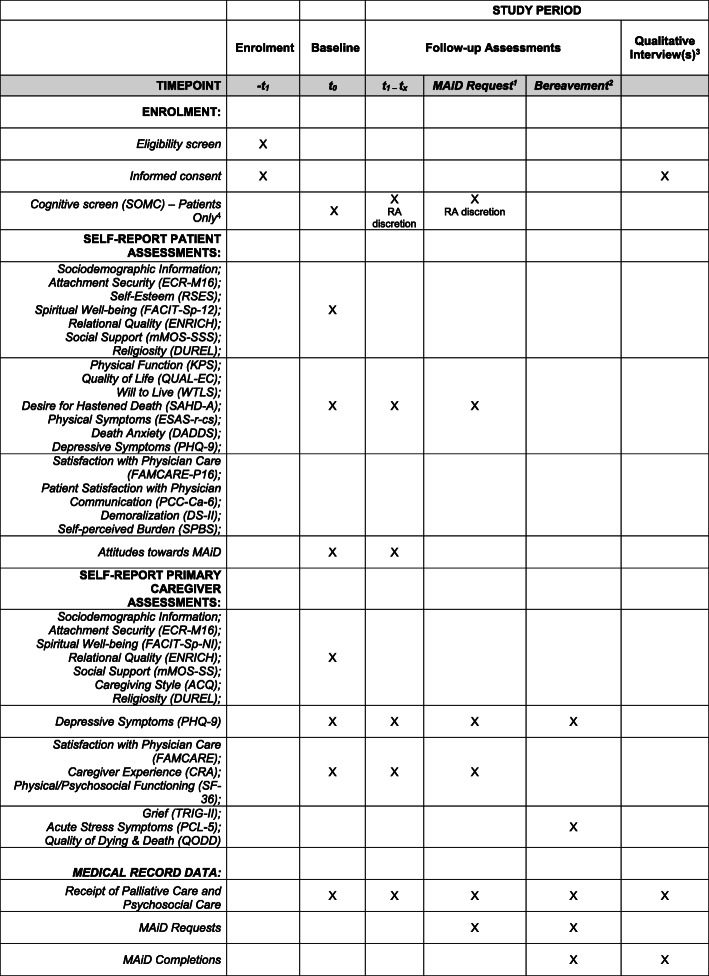
^1^If a patient requests MAiD during the study, even if it falls between the 6 monthly scheduled time points, the patient and their primary caregiver (if applicable), will be invited to complete a follow-up assessment and a qualitative interview at this time. ^2^In the event that a patient dies during the study (whether by MAiD or other causes), the patient’s primary caregiver (if applicable), will be approached within 6 months of the patient’s death to complete a final follow-up bereavement assessment and a qualitative interview. ^3^In addition to qualitative interviews with patients requesting MAiD and their participating caregivers, and bereavement interviews with participating caregivers whose loved ones die during the study, a subset of participants (patients and caregivers) will be identified through purposeful sampling and invited to complete one or more qualitative interview(s) at the 6 monthly follow-up time points over the course of the study. ^4^The SOMC may be re-administered at the discretion of the study research assistant if they feel the patient’s cognitive status may be impaired or have declined since the previous assessment. In the event that a patient fails a cognitive screen during the study, they will be withdrawn by the study principal investigator

At baseline, patients and caregivers will complete both sets of baseline-only and follow-up measures. All participants will provide sociodemographic information at baseline. The Short Orientation-Memory-Concentration Test (SOMC) [52] will be conducted with patients at baseline in order to test patient’s cognitive function; this measure may be repeated at follow-up at the discretion of the research assistant. See Tables 1 and 2 for a description of the key study measures listed below, and Fig. 2 for the schedule of participant assessments.

**Table 1:** Key Study Measures

Constructs and Measures	Descriptions
*Physical Function:* **The Karnofsky Performance Status index (KPS)** [53, 54]	Provides a valid and reliable rating in downward decrements of 10, from 100 (no signs/symptoms of illness) to 0 (death), of a patient’s level of physical functioning and ability to carry out activities of daily living. Administered by a member of the research staff with patient input. Modified to remove the 0 rating of “death” from the measure.
*Quality of Life*: **The Quality of Life at the End of Life Scale-Cancer scale (QUAL-EC)** [55, 56]	A 14-item, valid and reliable self-report scale that assesses quality of life in cancer patients near the end of life with four distinct factors: symptom control, relationship with healthcare provider, preparation for end of life, and life completion. Modified: Symptom control subscale has been removed [56].
*Will to Live:* **Will to Live Scale (WTLS)** [57]	A 5-item, self-report, validated and reliable instrument to assess the will to live.
*Desire for Hastened Death:* **The Schedule of Attitudes towards Hastened Death-Abbreviated (SAHD-A)** [58]	A 6-item, self-report, dichotomous measure of the desire for hastened death, validated in a large sample of patients with advanced illnesses.
*Depressive Symptoms: **Patient Health Questionnaire-9** **(PHQ-9)** [58, 59]	A 9-item, self-report, reliable and validated measure of depressive symptoms in patients with advanced cancer. Modified: One additional question (item 9a) added to assess intent to self-harm, which is answered only if item 9 assessing suicidal ideation is endorsed positively.
*Physical Symptoms:* **Edmonton Symptom Assessment System-Revised (ESAS-r-cs)** [60–62]	A revised, reliable and valid, 12-item version of the original 9-item ESAS that assesses the following common symptoms in advanced cancer and palliative care patients: pain, tiredness, drowsiness, nausea, lack of appetite, shortness of breath, depression, anxiety, and wellbeing. This recently modified version includes two additional symptoms (constipation and sleep disturbances), and the option to specify any other symptom.
*Attachment Security: **Experiences in Close Relationships-Modified (ECR-M16)** [63]	A modified 16-item, self-report, validated and internally reliable measure of attachment insecurity with two 8-item subscales of attachment avoidance and attachment anxiety.
*Self-Esteem:* **Rosenberg Self-Esteem Scale (RSES)** [64]	A widely-used, validated and reliable 10-item, self-report, measure of self-esteem.
*Death Anxiety:* **Death and Dying Distress Scale (DADDS)** [65–67]	A 15-item, self-report, validated measure of death anxiety in individuals with advanced cancer.
*Patient Satisfaction with Physician Communication:* **Patient-Centered Communication-Cancer-6 items (PCC-Ca-6)** [68]	A 6-item, valid and reliable self-report measure that assesses satisfaction with communication with doctors and other healthcare professionals such as nurses and physician assistants.
*Satisfaction with Physician Care: **Modified FAMCARE Scale P16 (FAMCARE-P16) for patients** [63] **and FAMCARE for caregivers** [69]	Reliable and valid self-report scales capturing satisfaction with physician care for patients [63] and for caregivers [69] including items evaluating assessments of information giving, availability of care, and physical care.
*Demoralization:* **Demoralization Scale-II (DS-II)** [70]	A 16-item, psychometrically sound self-report short-form version measuring the expression of demoralization. Contains two 8-item subscales; Meaning and Purpose, and Distress and Coping Ability.
*Self-perceived Burden*: **Self-perceived Burden Scale (SPBS)** [71]	A reliable and valid, 9-item self-report measure of chronically ill patients’ self-perceived experience of burden on their primary caregivers. Found to have a single factor, good reliability, and good convergent and divergent validity.
*Spiritual Well-being: **Functional Assessment of Chronic Illness Therapy-Spiritual Well-Being scale (FACIT-Sp-12) for patients** [72] **and FACIT-Sp-NI (Non-Illness version) for caregivers** [72, 73]	This widely used, reliable and valid 12-item self-report measure assesses the sense of meaning, peace, and faith in individuals with illness. Two items related to patient’s illness on the FACIT-Sp-12 were modified in the FACIT-Sp-NI (Non-Illness version of the scale) provided to caregivers (i.e., items 11 and 12 replace “illness” with “difficult times”).
*Relational Quality: **ENRICH Marital Satisfaction Scale (ENRICH)** [74]	This reliable and valid 15-item, self-report scale assesses satisfaction in various areas of the marital relationship and provided both dyadic and individual satisfaction scores. Patients not currently in a long-term romantic relationship will be instructed to skip this measure. Items referring to “marriage” were modified to refer to “relationship” to ensure relevance for non-married and common-law romantic partners.
*Social Support: **The modified Medical Outcomes Study Social Support Survey (mMOS-SSS)** [75, 76]	An 8-item self-report, shortened measure of social support with excellent psychometric properties [76], similar to the original 19 item scale [75]; the shortened measure provides scores for two subscales measuring emotional and instrumental (or tangible) social support.
*Religiosity: **The Duke University Religion Index (DUREL**) [77]	This is a 5-item, self-report, validated and reliable measure of three dimensions of religiosity: intrinsic religiosity (subjective religiosity), organizational religious activity, and non-organization religious activity. To promote inclusiveness, “Church” was modified to “places of worship” and “Bible Study” was modified to “study of religious texts”.

(*) signifies a measure that will be collected from both patients and primary caregivers

**Table 2:** Key Caregiver-Specific Measures

Constructs and Measures	Descriptions
*Caregiving Style:* **The Adult Caregiving Questionnaire** [78]	Reliable and valid 32-item, self-report scale that assesses four subscales representing four different patterns of caregiving: proximity, sensitivity, controlling, and compulsive. Modified: “Partner” was substituted with “relative/friend” to ensure suitability for all caregivers.
*Caregiver Experience:* **The Caregiver Reaction** **Assessment scale** [79–81]	A 24-item, self-report instrument that is feasible, reliable, and valid for assessing both negative and positive reactions to caregiving among partners of patients with cancer. Modified: A version was created substituting “partner” with “relative/friend” to ensure suitability for all caregivers.
*Physical and Psychosocial* *Functioning:* **The Medical Outcomes Study Short-Form 36 (SF-36)** [82]	A reliable and valid 36-item self-report measure assessing eight dimensions of functioning: physical functioning, role limitations owing to physical problems, role limitations owing to emotional problems, social functioning, mental health, general health perceptions, vitality, and bodily pain.
*Grief: **The Texas Revised Inventory of Grief-Part II (TRIG-II)** [83, 84]	The TRIG is a two-part questionnaire documenting past and present grief reactions. We will use Part II only, which has demonstrated reliability and validity and consists of 13 statements about present grief symptoms, including thoughts, feelings, memories, opinions, and attitudes.
*Acute Stress Symptoms: **The Posttraumatic Stress Disorder Checklist for DSM-5 (PCL-5)** [85, 86]	The PCL-5 is a 20-item, widely used DSM-5-correspondent self-report measure that assesses symptoms of Posttraumatic Stress Disorder (PTSD). The PCL-5 can be used to monitor symptoms, screen individuals for PTSD, and make a provisional PTSD diagnosis. It has been shown to be a measure that is valid, reliable, and useful in quantifying PTSD symptom severity.
*Quality of Death: **The Quality of Dying and Death questionnaire (QODD)** [87]	Administered by a research staff member/interviewer to bereaved proxy respondents, the QODD, the most widely used and best validated measure of the quality of death [88], asks caregivers about the patient’s last 7 days of life (if unconscious or unresponsive throughout the last 7 days, the focus is the last month before death) and covers 6 domains: symptoms and personal care, treatment preferences, time with family, whole person concerns, preparation for death, and moment of death.

(*) signifies a measure that will be collected only six months following a patient’s death

**Patient Baseline-Only Measures**: ***Attachment Security:*** Brief Modified Experiences in Close Relationships scale (ECR-M16) [89]; ***Spiritual Well-being:*** Functional Assessment of Chronic Illness Therapy-Spiritual Well-Being scale (FACIT-Sp-12, 58) ***Relational Quality:*** ENRICH Marital Satisfaction Scale (ENRICH) [74]; ***Social Support:*** The modified Medical Outcomes Study Social Support Survey (mMOS-SSS) [75, 76]; ***Religiosity:*** The Duke University Religion Index (DUREL) [77]; and, ***Self-Esteem***: The Rosenberg Self-Esteem Scale (RSES) [64].

**Patient Baseline and Follow-up Measures:** ***Physical Function:*** The Karnofsky Performance Status index (KPS) [53, 54]; ***Quality of Life:*** The Quality of Life at the End of Life Scale-Cancer scale (QUAL-EC) [55, 56]; ***Will to Live:*** The Will to Live Scale (WTLS) [57]; ***Desire for Hastened Death:*** The Schedule of Attitudes towards Hastened Death–Abbreviated (SAHD-A) [58]; ***Depressive Symptoms:*** Patient Health Questionnaire-9 (PHQ-9) [59]; ***Physical Symptoms:*** Edmonton Symptom Assessment System-Revised, including constipation and sleep disturbance (ESAS-r-cs) [60–62]; ***Death Anxiety:*** Death and Dying Distress Scale (DADDS) [65–67]; ***Patient Satisfaction with Physician Communication:*** Patient-Centered Communication-Cancer-6 items (PCC-Ca-6) [68]; ***Satisfaction with Physician Care:*** Modified FAMCARE Scale – Patient-16 items (FAMCARE-P16) [63]; ***Demoralization:*** Demoralization Scale-II (DS-II) [70, 79]; and, ***Self-Perceived Burden:*** Self-Perceived Burden Scale (SPBS) [71]. Participants will also answer several questions to assess their attitudes towards MAiD.

**Caregiver Baseline-Only Measures:** ***Attachment Security:*** Brief Modified Experiences in Close Relationships scale (ECR-M16) [89]; ***Spiritual Well-being:*** Functional Assessment of Chronic Illness Therapy-Spiritual Well-Being–Non-illness version (FACIT-Sp-NI) [72, 73]; ***Relational Quality:*** ENRICH Marital Satisfaction Scale (ENRICH) [74]; ***Social Support:*** The modified Medical Outcomes Study Social Support Survey (mMOS-SSS) [75, 76]; ***Caregiving Style:*** The Adult Caregiving Questionnaire [78]; ***Religiosity:*** The Duke University Religion Index (DUREL) [77].

**Caregiver Baseline and Follow-up Measures:** ***Depressive Symptoms:*** Patient Health Questionnaire-9 (PHQ-9) [59]; ***Satisfaction with Physician Care:*** FAMCARE Scale (FAMCARE) [69]; ***Caregiver Experience:*** The Caregiver Reaction Assessment scale [80]; ***Physical and Psychosocial Functioning:*** The Medical Outcomes Study Short-Form 36 (SF-36) [82].

We will also administer measures to caregivers six months post-patient death: ***Grief:*** The Texas Revised Inventory of Grief-Part II (TRIG-II) [83, 84]; ***Traumatic Stress Symptoms:*** The Posttraumatic Stress Disorder Checklist for DSM-5 (PCL-5) [85, 86]; ***Quality of Death:*** The Quality of Dying and Death questionnaire (QODD) [87]; and, ***Depressive Symptoms:*** Patient Health Questionnaire-9 (PHQ-9) [59].

***Chart Review Form:*** Patients’ medical records will be reviewed at baseline and at each follow-up in order to document: i) medical diagnosis, date of diagnosis, and current cancer stage; ii) medical history including current comorbid medical and psychiatric diagnoses; iii) treatments received, including the timing of psychosocial oncology and/or palliative care interventions; and iv) emergent DD/suicidality events. We will obtain from the MAID clinical database the reasons of the patient and/or assessor-rated reasons for MAiD requests, MAiD approvals, MAiD completions, and non-completions.

***Qualitative Interview:*** A semi-structured interview will be used. This interview guide will be further developed and reviewed as the constant comparative analysis progresses [90]; emerging themes in earlier interviews will serve to refine interview questions and probes. Open-ended enquiry will be made in the initial interview to understand the illness experience, including; caregiver stress, burden, support needs and support received, emotional and physical distress; attitudes about MAiD and advance directives. All interviews will be audiotaped, transcribed verbatim, verified and de-identified prior to analysis.

### Data collection procedures

#### Baseline assessment

Participants will be given the option of taking the questionnaire package home and returning the completed package in a self-addressed stamped envelope or completing it online. Participants will also be provided the option of having the questions read to them by a member of the research team as some participants may require this assistance due to their state of health. Medical data will be extracted from review of the medical record of each patient.

#### Longitudinal follow-up assessments

Participants will complete follow-up questionnaires every six months until study completion, unless they voluntarily withdraw, are unable to participate due to impairment in cognitive or physical functioning or die. An additional assessment will be made at the time of a MAiD request. Following the assessment, the medical record review will be updated. Where applicable, the date of death will be obtained from the medical record. In the case of patient death, participating primary caregivers will be followed-up approximately six months after this event to complete a bereavement assessment.

#### Qualitative assessments

Qualitative interviews will be conducted in a subset of the study participants. A quota sampling method [51] will be used to select a subset of patients and primary caregivers for one or more qualitative interview(s), conducted at six monthly intervals. Purposeful sampling will be based on high and low outcomes on the Schedule of Attitudes towards Hastened Death-Abbreviated (SAHD-A) (for patients) [58], caregiving burden and distress measures (for caregivers), and sociodemographic representation (for both patients and caregivers). We estimate that the saturation point will be reached at 10–15 participants per group, but we will recruit participants until saturation is reached. A grounded theory approach with constant-comparative analysis [90] will be used to identify themes and to determine the sample size at saturation point [91]. The interviews will last up to 60 min, depending upon the participants’ ability to participate, and will be conducted by a trained interviewer at the convenience of the participants over the telephone, in the clinic, or using a UHN-approved online platform. A frequency of six-monthly intervals was chosen in order to minimize participant burden but still capture as much of their longitudinal experience as possible. Enquiry will be made in follow-up interviews about issues raised in the first interview. Should a participating patient request MAiD, they, and their primary caregiver (if applicable), will be invited to complete an interview at that time, even if it falls between scheduled time points. Should a participating patient die during the study, either from MAiD or other causes, their primary caregiver (if applicable) will also be invited to complete one final semi-structured interview approximately six months after the patient’s death.

### Data analysis

#### Survey data

Descriptive statistics, desegregated by sex and gender, will be calculated to provide summary information about the characteristics of the participants at baseline. For each assessment time, descriptive statistics will be calculated for the DD outcomes and all other predictive variables. Prevalence of the DD, as measured by the SAHD-A, will be compared to our historical dataset [92]. The distributions of the continuous variables will be examined for non-normality, and transformations will be employed where necessary. Bivariate associations between DD variables and baseline medical and sociodemographic factors, as well as associations of these baseline variables with physical, psychosocial, and psychological risk factors will be described by calculating subgroup means and standard deviations for categorical variables and correlation coefficients for continuous variables.

The analysis of the longitudinal data will be conducted using mixed linear models (MLM), [93] which can take into account incomplete data and both time-varying and time-invariant predictors. To accommodate anticipated variations in individual profiles, random effects models will be fitted. MLM logistic regression will be used to determine predictors of both MAiD requests and completions. Similar exploratory analyses will be conducted to identify associations with emergent suicidality. We will replicate the MLM analysis from our previous study of the DD, [8] now incorporating receipt (or not) of psychosocial and/or palliative care interventions, and death anxiety as additional predictors of the DD (Fig. 1). To examine the statistical significance of interactions among medical, sociodemographic, physical, and psychosocial factors as suggested by the exploratory analyses, relevant interaction terms will be included in the MLMs. To avoid over-fitting the model, only those interactions regarded as clinically relevant will be tested in the model.

#### Qualitative interviews

A grounded theory approach will guide the qualitative data collection and analysis [94]. Conceptual categories and themes for coding will be derived directly from analyzing the interview material, with a view to inspecting deviant or negative cases in order to refine emerging propositions or hypotheses. This iterative process will continue until theoretical saturation is reached. The data analysis will use an inductive, constant comparison method to code or index the data. Once all data that matches that theme have been located, the process will be repeated to identify further themes or categories. Categories will be added to reflect the nuances in the data as possible. Cross-indexing will allow for the analysis of data items that fit into more than one category. Systematic comparative analysis will be used to identify differences and similarities between men and women, and those with and without a high DD (for patients), and with and without high caregiving burden and/or distress (for caregivers). Categories will also be examined in a longitudinal manner to identify patterns of emergence and development of categories over time and these finding will be compared to the quantitative data, particularly for the impact of goals of care conversations on desire for MAiD. Our analysis will be informed by our previous qualitative study on the DD [12].

## Discussion

### Methodological strengths

This project is a longitudinal study of the course, predictors, and nature of the desire for MAiD in patients with advanced cancer in a geographic setting where MAiD is legally available. It includes quantitative and qualitative methods and uses a comprehensive battery of validated scales to measure the physical and psychosocial variables that we hypothesize are associated with the DD and MAiD requests and completions. The large study sample of patients and caregivers is a strength of the study design, enabling the evaluation of hypotheses in sub-groups. The study also will collect a wide range of sociodemographic information, which will enable the evaluation of the social determinants of the DD and MAiD, as well as examination of health equity in the receipt of MAiD. The quality of the research data collected will be ensured by having experienced, trained research personnel follow best research practices and institutional guidelines, including the use of standardized source notes and documentation of study participation in the patient’s clinical research record, to ensure rigour in the data collection processes. Research personnel will employ direct online data entry whenever possible; duplicate data entry for questionnaires completed in hard copy; and verification of transcribed interviews and all data extracted through medical chart review (e.g., disease and treatment-related data, supportive care services data, MAiD clinical data). Processes for confidentiality and secure storage of data have been ensured through REB approval, and data will be retained for 10 years post study completion as per REB guidelines at our institution. Further, all studies at PM are subject to internal audit by the Cancer Clinical Research Unit and/or the UHN Research Quality Integration teams, both of whom are independent of the study team.

### Foreseeable limitations and mitigation strategies

The main challenge of this study is the requirement to recruit a large sample of patients with advanced or metastatic cancer and their primary caregivers. However, in our previous research experience with this population, we found that a 60% recruitment rate is achievable, without adverse effects from completion of a battery of measures of this kind [8]. Although there are a relatively large number of measures, we have minimized participant burden by limiting the number of measures administered longitudinally, selecting measures that are relatively brief or have been shortened from their original versions, offering the option of printed copy and online questionnaire completion at the hospital or at home, providing assistance with completion of questionnaires as requested in-person, online, or over the telephone, and conducting the qualitative interviews at the participants’ convenience. Participants with significant suicidality, physical or emotional distress identified by the research staff will be offered referral for psychosocial oncology and palliative care services in the Department of Supportive Care at PM, with notification to the attending oncologist with the patient’s consent. For caregivers (including bereaved caregivers) who express significant distress when completing the measures or qualitative interviews, a referral to the Department of Supportive Care’s Caregiver Clinic at PM for bereavement and/or psychosocial support will be offered.

### Knowledge translation

Integral to the goals of this study is knowledge translation in the form of training healthcare providers to respond appropriately to expressions of the DD from patients in this new era of MAiD. Our findings will be directly incorporated into UHN MAiD training workshops, invited speaker engagements and the UHN MAiD website, to inform MAiD teams. Dissemination will occur through publications and conference presentations locally, nationally, and internationally, and as well as via the website of the Global Institute of Psychosocial, Palliative and End-of-Life Care (GIPPEC; see www.gippec.org) and the Canadian Association of MAiD Assessors and Providers (CAMAP; see camapcanada.ca), thus bridging the knowledge to action gap.

### Study implications

Data from this study will contribute significantly to our understanding of the DD in a setting where MAiD is legal, and inform critical questions regarding the utility of advance directives for MAiD, the management of depression in MAiD assessment, and how to guide healthcare providers in responding to DD statements with advanced cancer patients. The findings from this study will inform the training of oncologists and other healthcare providers in conversations about the DD and MAiD, and the assessment for MAiD eligibility through identifying distinctions between the DD, depressive suicidality, and the desire for MAiD in patients with advanced cancer. The results will also provide an evidence base to inform proposed changes in MAiD legislation, including the potential permissibility of MAiD in mental illness and as an advance directive.

## Data Availability

The datasets to be generated and/or analysed during the current study will not be publicly available due to institutional privacy and confidentiality guidelines. Additional information may be available from the corresponding authors on reasonable request.

## Abbreviations

CRA: Caregiver Reaction Assessment scale
DADDS: Death and Dying Distress Scale
DD: Desire for Death
DHD: Desire for Hastened Death
DS-II: Demoralization Scale-II
DSM-5: Diagnostic and Statistical Manual for Mental Disorders, 5th Edition
DUREL: Duke University Religion Index
ECR-M16: modified Experiences in Close Relationships scale
ENRICH: ENRICH Marital Satisfaction Scale (ENRICH)
ESAS-r-cs: Edmonton Symptom Assessment System-Revised (including constipation and sleep disturbances)
FACIT-Sp-12: Functional Assessment of Chronic Illness Therapy-Spiritual Well-Being scale
FACIT-Sp-NI: Functional Assessment of Chronic Illness Therapy-Spiritual Well-Being-Non-Illness version
FAMCARE-P16: Modified FAMCARE Scale for Patients
KPS: Karnofsky Performance Status index
MAiD: Medical Assistance in Dying
MLM: Mixed Linear Models
mMOS-SSS: modified Medical Outcomes Study - Social Support Survey
PCC-Ca-6: Patient-Centered Communication-Cancer-6 items
PCL-5: Posttraumatic Stress Disorder Checklist for DSM-5
PHQ-9: Patient Health Questionnaire-9
PM: Princess Margaret Cancer Centre
QODD: Quality of Dying and Death questionnaire
QUAL-EC: Quality of Life at the End of Life – Cancer scale
SAHD-A: Schedule of Attitudes towards Hastened Death-Abbreviated
SOMC: short Orientation-Memory-Concentration Test
SPBS: Self-Perceived Burden Scale
TRIG-II: Texas Revised Inventory of Grief – Part II
UHN: University Health Network
WTHD: Wish to Hasten Death
WTLS: Will to Live Scale.

## Footnotes

1 The percentage of total cancer-related deaths in Canada was calculated by dividing MAID-related cancer deaths in 2020 (5,248) [4] by total cancer deaths in 2020 (83,300) [7].
